# Lateral Ramus Cortical Bone Plate in Alveolar Cleft Osteoplasty with Concomitant Use of Buccal Fat Pad Derived Cells and Autogenous Bone: Phase I Clinical Trial

**DOI:** 10.1155/2017/6560234

**Published:** 2017-12-12

**Authors:** Arash Khojasteh, Lida Kheiri, Hossein Behnia, Azita Tehranchi, Pantea Nazeman, Nasser Nadjmi, Masoud Soleimani

**Affiliations:** ^1^Department of Oral and Maxillofacial Surgery, Research Institute of Dental Sciences, School of Dentistry, Shahid Beheshti University of Medical Sciences, Tehran, Iran; ^2^University of Antwerp (UA), Antwerp, Belgium; ^3^School of Dentistry, Shahid Beheshti University of Medical Sciences, Tehran, Iran; ^4^Department of Orthodontics, Research Institute of Dental Sciences, School of Dentistry, Shahid Beheshti University of Medical Sciences, Tehran, Iran; ^5^Research Institute of Dental Sciences, School of Dentistry, Shahid Beheshti University of Medical Sciences, Tehran, Iran; ^6^OMFS at the University of Antwerp, Antwerp, Belgium; ^7^The Team for Cleft & Craniofacial Anomalies, Antwerp, Belgium; ^8^Department of Hematology, Tarbiat Modares University, Tehran, Iran

## Abstract

Tissue regeneration has become a promising treatment for craniomaxillofacial bone defects such as alveolar clefts. This study sought to assess the efficacy of lateral ramus cortical plate with buccal fat pad derived mesenchymal stem cells (BFSCs) in treatment of human alveolar cleft defects. Ten patients with unilateral anterior maxillary cleft met the inclusion criteria and were assigned to three treatment groups. First group was treated with anterior iliac crest (AIC) bone and a collagen membrane (AIC group), the second group was treated with lateral ramus cortical bone plate (LRCP) with BFSCs mounted on a natural bovine bone mineral (LRCP+BFSC), and the third group was treated with AIC bone, BFSCs cultured on natural bovine bone mineral, and a collagen membrane (AIC+BFSC). The amount of regenerated bone was measured using cone beam computed tomography 6 months postoperatively. AIC group showed the least amount of new bone formation (70 ± 10.40%). LRCP+BFSC group demonstrated defect closure and higher amounts of new bone formation (75 ± 3.5%) but less than AIC+BFSC (82.5 ± 6.45%), suggesting that use of BFSCs within LRCP cage and AIC may enhance bone regeneration in alveolar cleft bone defects; however, the differences were not statistically significant. This clinical trial was registered at clinicaltrial.gov with NCT02859025 identifier.

## 1. Introduction

The most common facial congenital malformation, cleft lip and palate, can disturb patient functions and cause psychological problems [1, 2]. Failure of fusion between the nasal process and oropalatal shelves leads to alveolar cleft in 0.36 to 0.83 out of every 1000 live births. Seventy-five percent of all cleft lip and palate variations are accompanied by alveolar bone defects [3–5]. Despite the psychological benefits of secondary alveolar bone grafting, it is also necessary for maxillary arch integration, easier teeth eruption, alar base support, closure of oroantral communication, and increasing quality of life [5–7]. Due to the great amount of corticocancellous bone in iliac crest, it is the most popular donor site for harvesting autogenous bone [8]. Mandibular lateral ramus provides an accessible intraoral cortical bone and is routinely administered in preimplant ridge augmentation [9, 10] but the amount is limited comparing to the iliac crest [11] although long term hospitalization (specifically for iliac crest harvesting), pain, donor site morbidities, and great cost [12] are the primary disadvantages of alveolar cleft autografting [12].

To overcome the aforementioned shortcomings, tissue engineering was developed as an alternative promising approach by merging the fields of cell biology, biomaterial engineering, and medicine to fabricate personalized functional tissues [8]. It reduces donor site morbidity, postoperative pain, inadequate bone regeneration, additional cost, and hospitalization [13]. Mesenchymal stem cells (MSCs) are multipotential cells that can be harvested from mesodermal tissues such as bone marrow, dental pulp, periosteum, and fat [14] and they have demonstrated some degree of bone healing in various defects [6, 15]. Adipose-derived stem cells (ASCs) are widely utilized in tissue engineering for several years. Their advantage over other sources is that they are usually obtained from disposable tissues of liposuction and some studies have found their properties comparable to bone marrow derived stem cells (BMMSCs) [16–18]. Buccal fat pad (BFP) is an adipose tissue with a rich source of MSCs [19]. This source has been in the spotlight since it may be harvested with an intraoral surgery with minimal morbidity and discomfort [20] and also craniofacial sources may provide a better niche for regenerative techniques in oral and maxillofacial areas [14].

Growth factors and MSCs are two major approaches for bringing tissue engineering from the bench to the bedside [13, 21] and combination of MSCs with various growth factors such as recombinant human bone morphogenetic protein-2 (RhBMP-2) and platelet rich plasma has been utilized in this context [8]. Several efforts are made to treat alveolar bone defects with the combination of BMMSCs [13] and growth factors [6]. However, the amount of generated bone was not comparable to the gold standard, the autogenous bone graft [6, 13].

A wide range of data and lack of predictability have led to a continuing search for better results. Cell sources, differentiation protocols, incubation periods, passage counts, types of scaffolds, and delivery methods varied between studies [22].

Recently we published the effectiveness of the concomitant use of buccal fat pad derived stem cells (BFSCs) with the autogenous iliac bone in the treatment of maxillomandibular atrophy [20]. In the present study, our aim was to combine regenerative techniques with bone grafting in human alveolar cleft models to increase effectiveness and survival of reconstructed tissue. The MSCs in this study were derived from BFP and cultured over natural bovine bone mineral granules (NBBM) and delivered within the lateral ramus cortical bone plate (LRCP) or anterior iliac crest (AIC).

## 2. Materials and Methods

### 2.1. Patients

**Figure 1:**
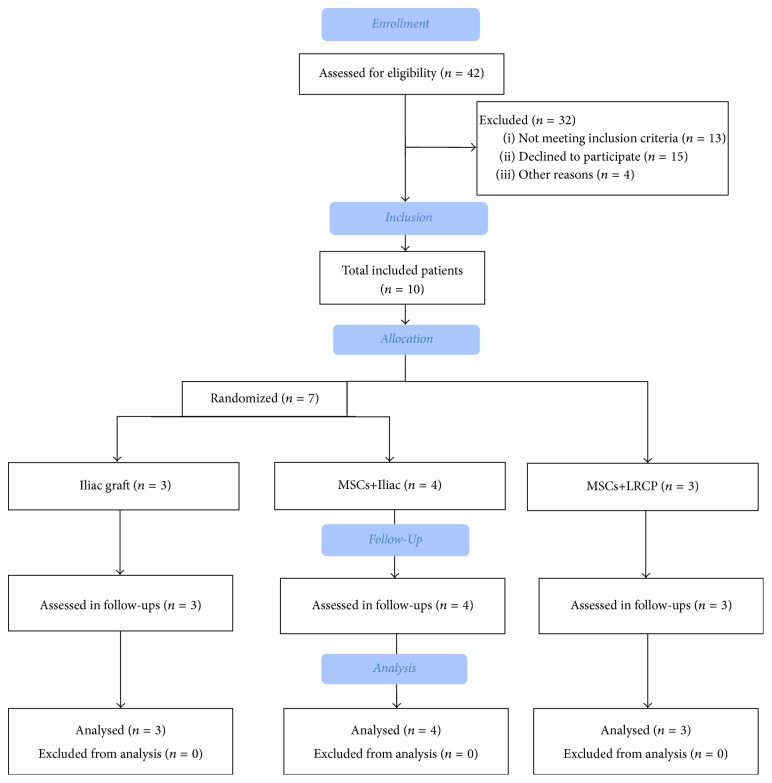
Consort flow diagram demonstrating study protocol.

This study was a prospective randomized clinical trial. The inclusion criteria for this study were possession of unilateral cleft lip and palate, receipt of presurgical orthodontic treatment, and performance of secondary alveolar bone grafting as the only remaining surgical procedure. The patients were excluded if they had any systemic disorder interfering with the surgery and healing. From the patients suffering from cleft lip and palate referred to Department of Oral and Maxillofacial Surgery at Taleghani Hospital, Tehran, Iran, in 2015, ten patients (3 females) with unilateral cleft lip and palate met the criteria and were included in this study (Figure 1). Four patients were adults and therefore had lost the chance for canine eruption. All others were aged 8 to 14 years and had concomitant growing teeth in the cleft space. Computed tomography was used to assess the defect location and size. Permission was obtained to complete the procedure despite the absence of well-documented data supporting stem cell-induced bone regeneration in cleft patients. All procedures and study protocols were approved by the institutional ethical committee at Shahid Beheshti University of Medical Sciences (ClinicalTrials.gov identifier: NCT02859025), and informed consent was obtained from all the patients. Isolation and cultivation of the MSCs were performed without xenogenic supplements such as fetal calf serum to avoid immune reaction. All methods used to cultivate and extract MSCs were as described in previous projects using human patients [6, 13, 20]. Patients were allocated to 3 treatment groups. In the first group (3 patients) treatment was performed by AIC spongy bone to fill the defects, followed by coverage with collagen membrane (Jason membrane; Botiss Biomaterials GmbH, Berlin, Germany) (the control group, AIC group).

Adult patients were selected for the second group (3 patients) as harvesting a LRCP of the appropriate size requires a mature ramus with erupted molars.

Accordingly, LRCP was used to create a protected healing space by fixing it to adjacent walls of the cleft defect. BFSCs were loaded onto NBBM (Cerabone; Botiss, Berlin, Germany) and delivered to the defect (LRCP+BFSC group). In the third group 4 patients were treated with AIC as in the control group, but BFSCs were cultured over NBBM and put over the spongy bone and covered with a collagen membrane (AIC+BFSC group).

### 2.2. Isolation of MSCs from the BFP

**Figure 2:**
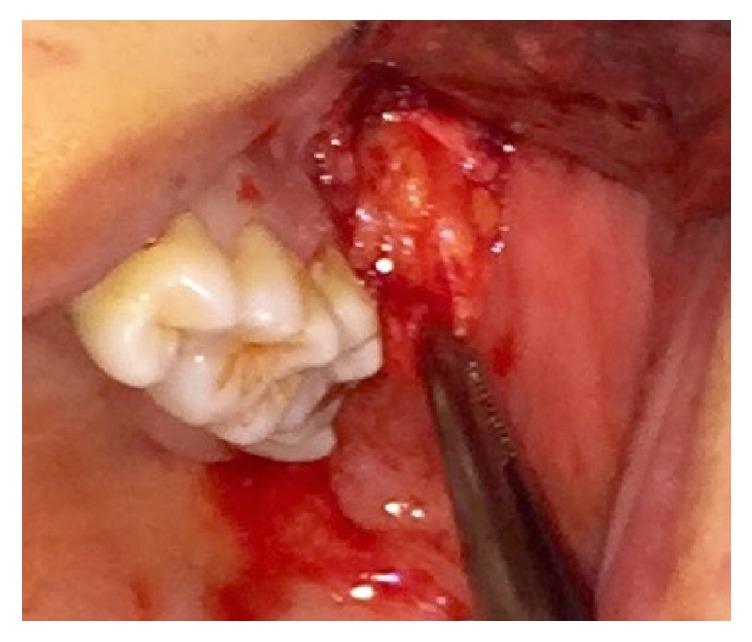
Buccal fat pad harvesting. Buccal fat pad was exposed and harvested using a vestibular incision distal to the second molar.

**Figure 3:**
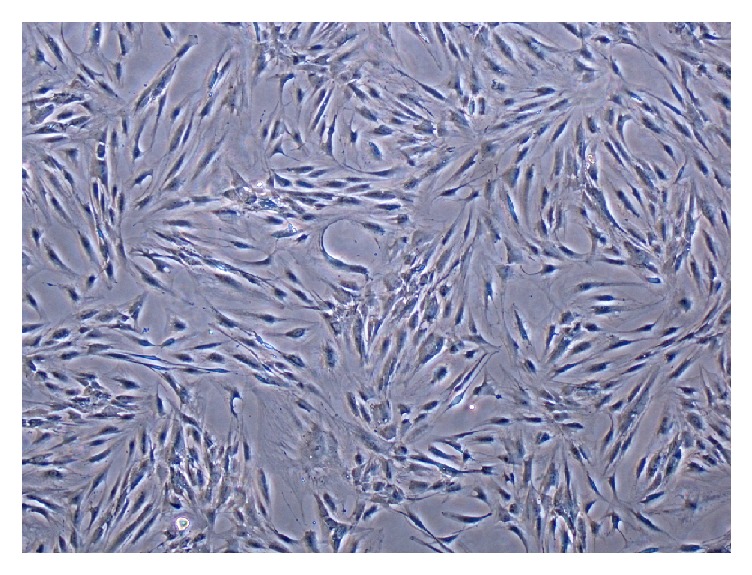
Light microscopic evaluation of the stellate like cells extracted from buccal fat pad.

Isolation of adipose-derived MSCs from BFP was performed according to our previous published protocol [20]. The BFP tissues were harvested from healthy donors through a vestibular incision distal to the maxillary second molar (Figure 2). Tissues were exposed with a blunt dissection while preserving the thin covering membrane. 3 to 5 ml of BFP was excised and delivered to the lab in DMEM medium. Dissected tissues were minced and washed with phosphate buffered saline (PBS) twice. Then, they were incubated in 3 mg/ml type I collagenase in PBS (GIBCO Laboratories, Grand Island, NY, USA) at 37°C for 30 min. The cell suspensions were centrifuged to make cell pellets. The pellets were then resuspended in *α*-MEM culture medium supplemented with 10% human serum and placed in 25-cm^2^ culture flasks followed by incubation in a humidified atmosphere containing 5% CO_2_ at 37°C. Culture media were changed twice weekly and, after reaching the 85% confluence, the cells were removed by enzymatic digestion (0.25% trypsin–EDTA) and passaged. MSCs of the third to fourth passage were subjected to the experiments [20]. Adherent cells were expanded as monolayer cultures in a 95/5 air/CO_2_ (v/v) atmosphere at 37°C with media changes every 3 days. 85% confluent cells were dissociated with trypsin and subcultured in new 6-well culture dishes at a plating density of 6 × 10^4^ cells/dish. The cells were monitored daily under light microscopy (Figure 3).

#### 2.2.1. Preparation of Human Serum

From each patient, 20 ml whole blood was drained into blood bags (Baxter, Deerfield, IL), quickly transferred to 10-ml Vacutainer® tubes without anticoagulants (BD, Plymouth, UK), and allowed to clot for 4 h at 4 to 8°C. Subsequently, the blood was centrifuged at 1800*g* at 4°C for 15 min. Serum was collected and filtered through a 0.2-mm membrane (Sarstedt, Nümbrecht, Germany). Aliquots of the sterile serum were stored at −20°C. The lab process and cultivation of cells from each patient were performed without significant salience [6].

### 2.3. Evaluation of the MSC Nature of the Isolated Cells

#### 2.3.1. Surface Marker Analysis

Fluorescent isothiocyanate (FITC)-conjugated monoclonal antibodies were applied to the isolated cells using the flow cytometry device (BD FACS Calibur, Franklin Lakes, NJ) to measure cell surface markers expression. To do this, cells at passage 3 were harvested and resuspended in PBS at concentration of 10^5^ per sample stained for 30 min at 4°C in the dark room with antibodies against human anti-CD44-FITC, anti-CD90-FITC, anti-CD73-PE, anti-105-PE, anti-CD45-FITC, and anti-CD34-PE (EXBIO, Vestec, Czech Republic) which were used at 2 *μ*g/ml. Specimens containing at least 90% fluorescent-labeled cells were regarded as positive. The differentiation potential towards the osteogenic and adipogenic lineages was examined for the stemness of the cultured cells. Third passage cultured cells were plated at a cell density of 10^5^ cells/well at 24-well plates and treated with osteogenic medium containing 10 mmol *β*-glycerol phosphate (Sigma-Aldrich, St. Louis, MO or Taufkirchen, Germany), 50 *μ*g/ml ascorbic-2-phosphates (Sigma), and 10^−7^ M dexamethasone (Sigma) for 14 days. Subsequently, to verify osteogenic differentiation, stem cells were fixed with 4% paraformaldehyde for 10 min and stained with alizarin red. Adipogenic medium containing DMEM with 0.5 mM 3-isobutyl-1-methylxanthine (Sigma-Aldrich, St. Louis, MO or Taufkirchen, Germany), 250 nM dexamethasone, and 0.2 mM indomethacin was also used to induce adipogenic differentiation for 14 days in third passage cells. Cells were fixed using 4% paraformaldehyde, washed with 70% ethanol, and stained using oil red solution in 99% isopropanol for 15 min [6].

### 2.4. Implant Preparation

In all cases, 3 days before transplantation, implants were loaded with the cells obtained from the third subculture. 10^6^ cells were loaded on 2 ml Cerabone (Botiss, Berlin, Germany) which is a granular biomaterial with a 200 to 850 *μ*m particle size.

#### 2.4.1. Scanning Electron Microscopic Analysis

**Figure 4:**
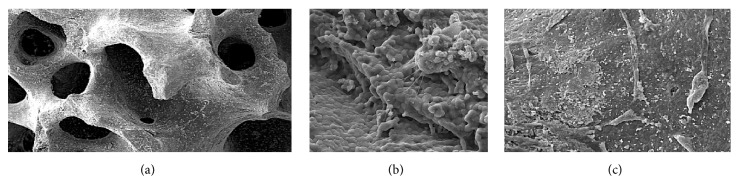
(a) SEM evaluation views of NBBM granule (×50); (b) BFSCs laid down into the scaffold through cellular pods and attachments at ×500 and ×1000 view (c).

Cell-loaded scaffolds were fixed in 2.5% glutaraldehyde (Merck KGaA, Darmstadt, Germany), washed with PBS, dehydrated in graded ethanol, vacuum dried, and coated with gold. The prepared block was examined under a scanning electron microscope (Hitachi, Tokyo, Japan). MSCs were scattered within the pores of the scaffold. Adherence to the scaffold was demonstrated by cellular pods and attachments (Figures 4(a)–4(c)).

### 2.5. Surgical Procedure

**Figure 5:**
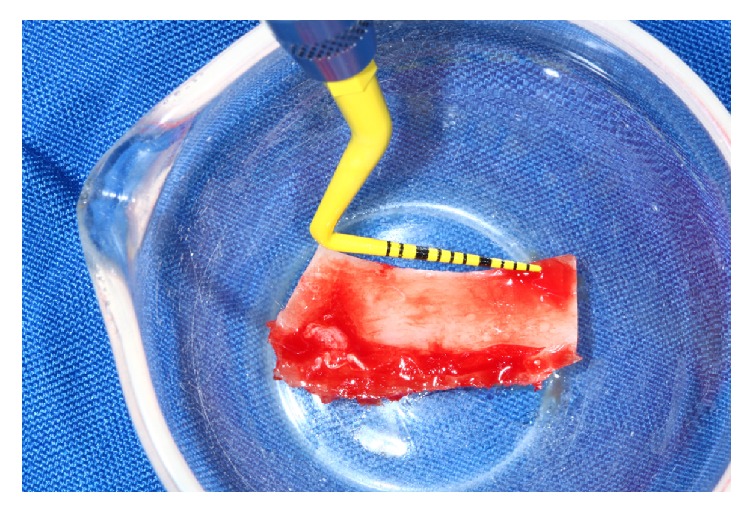
Lateral ramus cortical bone was harvested from lateral side of the mandible.

**Figure 6:**
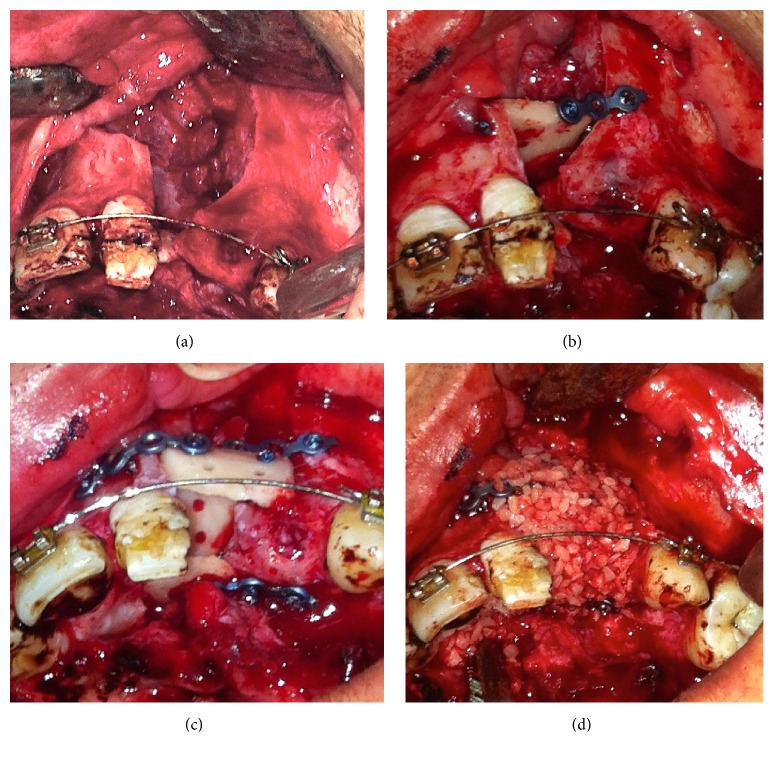
Preparation and placing of LRCP; LRCP was trimmed and cut to 2-3 pieces and fixed in defect region. Protected healing space was created and BFSCs were delivered to the space.

**Figure 7:**
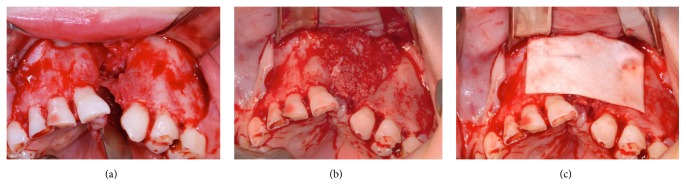
Anterior iliac crest spongy bone was filled in the alveolar defect in control group and covered with collagen membrane.

**Figure 8:**
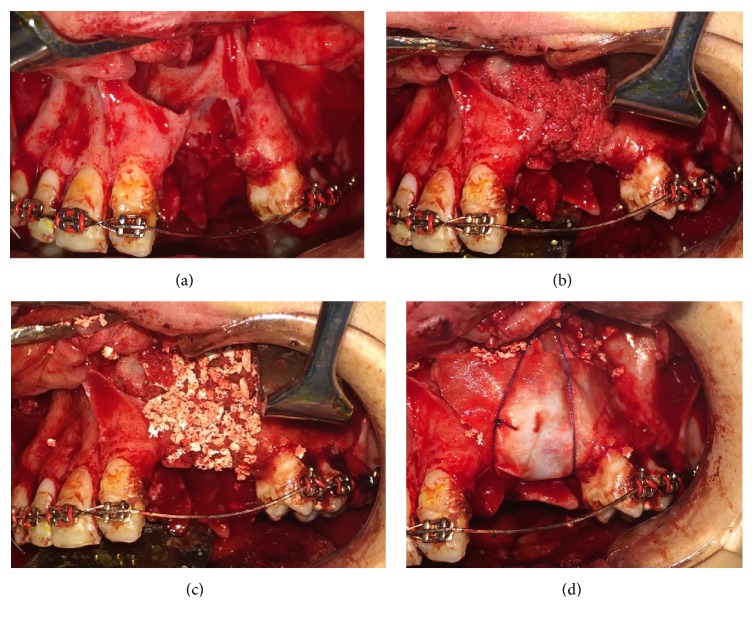
Scaffolds carrying BFSCs covered the spongy iliac bone in AIC+BFSC group.

Surgery was performed under general anesthesia. Following a crestal incision at the level of the gingival sulcus, dissections were made in the scar tissue to reach the bony surface of the cleft walls. The tissue was then elevated beneath the periosteum plane to the level of the anterior nasal spine anteriorly, the lateral piriform rim superiorly, and the alveolar ridges inferiorly. The flaps of the nasal floor and the oral mucosa formed the ceiling and floor of the cleft cavity, respectively. Concomitant injection of Cefazolin (1 g) was used during the perioperative period and followed by a 3-day course. In LRCP+BFSC group, LRCP was harvested from one or both sides of the mandibular ramus according to the defect size (Figure 5). Lateral ramus cortical bone was trimmed and split into 2-3 pieces to create a protected healing space in the alveolar cleft defect. The mixture of scaffolding and cells was transferred to the defect using microforceps (Figures 6(a)–6(d)). The wound was subsequently closed in a water-tight tension-free manner. In AIC group, cleft defects were treated with AIC spongy bone by placing the cortical bone in the nasal floor and covering with a collagen membrane (Figures 7(a)–7(c)). In AIC+BFSC group, cell-loaded NBBM was put over the AIC spongy bone and covered with collagen membrane (Figures 8(a)–8(d)).

#### 2.5.1. Clinical Evaluation

Soft tissue healing and normal healing sequences of grafted tissues were evaluated every 2 weeks.

#### 2.5.2. Radiographic Evaluation

Cone beam computed tomography was obtained after 6 months. The outline of 1-mm coronal sections of the treated region was taken before and after surgery, and new bone formation was assessed. The outlined sections of the alveolar defect were used to determine the preoperative defect, postoperative defect, and volume of bone fill using Image Pro software (National Institutes of Health [NIH], Bethesda, MD).

#### 2.5.3. Data Analysis

All statistical analyses were performed using a software package (SPSS Statistics version 20.0, IBM Corp, Armonk, NY). The level of bone formation was compared between the groups by nonparametric ANOVA test. A significance level of 0.05 was used for all comparisons.

## 3. Results

### 3.1. In Vitro Assessment

**Figure 9:**
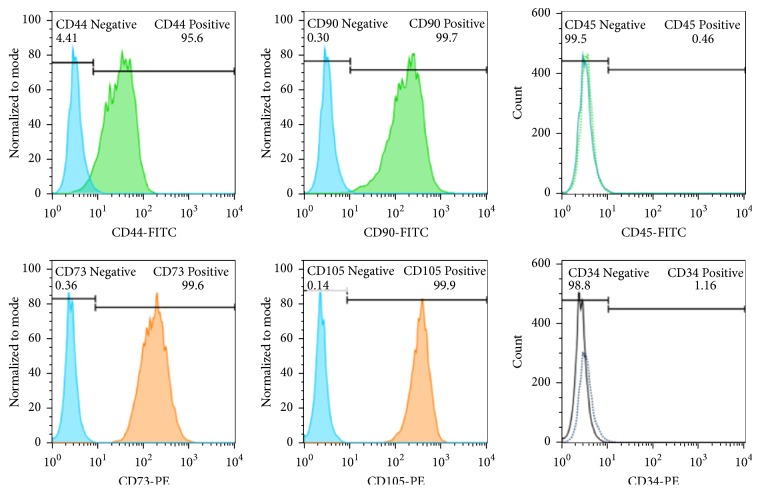
Flow cytometeric evaluation of the human buccal fat pad derived stem cells. CD 70, 93, 44, and 105 were detected positive and CD 45 and 34 were negative.

**Figure 10:**
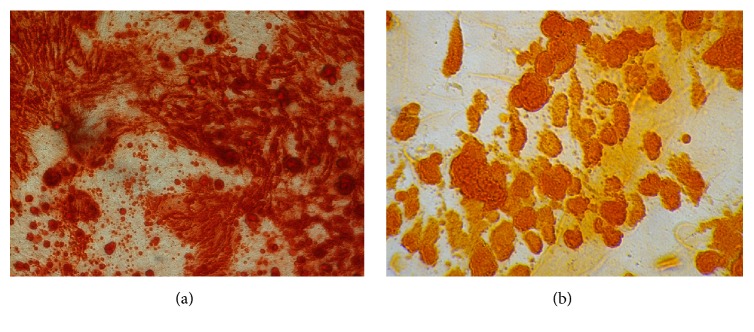
(a) Alizarin red staining. Nodule-like structures of mineralized matrix were observed under inverted light microscope. (b) Oil red staining revealed positive result for in vitro adipogenic differentiation.

Assessment by flow cytometry device demonstrated that more than 95% of the cells were positive for CD44, CD90, CD73, and CD105, MSC-surface markers, whereas they were negative for CD45 and CD34, hematopoietic markers (Figure 9). For multilineage differentiation, the cells were evaluated by inverted light microscopy at 2 weeks postculture and they had differentiated into either osteogenic or adipogenic lineage (Figures 10(a) and 10(b)).

### 3.2. Clinical Assessment

**Figure 11:**
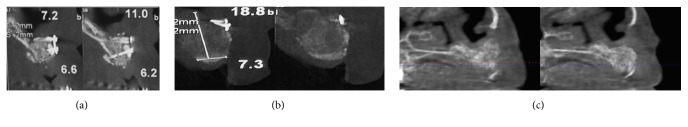
Tomography of new bone formation in LRCP+BFSC. (b) Tomography of new bone formation in AIC+BFSC group. (c) Radiographic investigation of new bone formation in control group.

Successful healing with no fistula or oronasal communication was achieved in all cases except one patient developed partial dehiscence which was managed by instructing oral hygiene and prescribing mouthwash. One case in LRCP+BFSC group showed partial exposure of lateral ramus cortical bone in the labial side. Radiomorphometric values for new bone formation in cleft defects are shown in Table 1. After 6 months, members of LRCP+BFSC experienced between 69% and 85% new bone formation (BF) (Figure 11(a)) while, for those in AIC+BFSC group, it was 70%, 85%, and 90% (Figure 11(b)). The controls (AIC group) experienced 65%, 70%, and 85% new bone (Figure 11(c)). The mean new bone formation was highest in AIC+BFSC, but not at a statistically significant level (*p* > 0.05).

### 3.3. Histological Assessment

**Figure 12:**
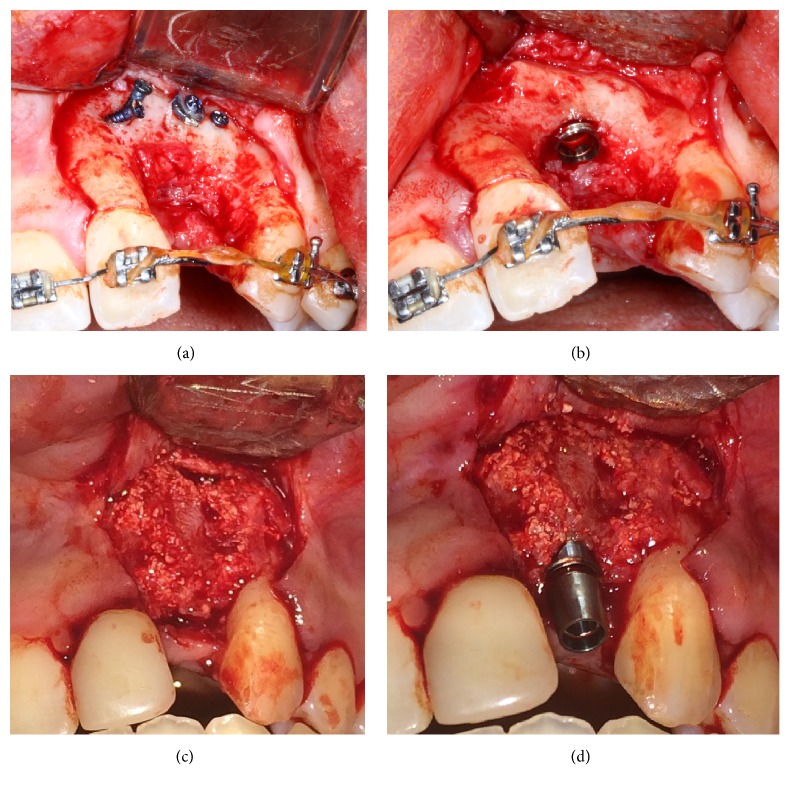
(a) Bone Healing after 9 months in LRCP group. (b) Dental Implant placement in new regenerate bone. (c) Healed alveolar cleft defects in AIC+BFSCs group. (d) Dental implant placement in new regenerate bone.

**Figure 13:**
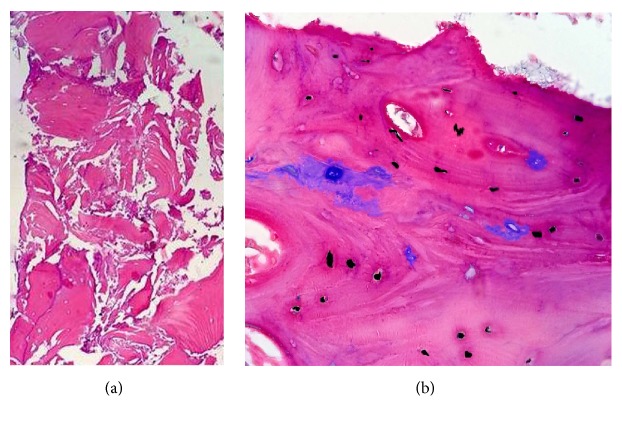
Normal bone with active osteoblasts producing osteoid matrix in the presence of a fibrous type marrow (H&E ×10). (a) Hematoxylin & eosin staining ×10, (b) ×40.

We placed a dental implant in one patient from LRCP group and one from AIC+BFSC group. The new regenerated bone in the LRCP healing space appeared healthy with adequate stability during drilling sequence (Figures 12(a)–12(d)). A two millimeter trephine biopsy was taken from the surgical site and histological analysis was performed following hematoxylin and eosin staining. Histological analysis showed new lamellar bone with osteoblastic rim without inflammatory cells infiltration (Figures 13(a) and 13(b)).

## 4. Discussion

Despite the benefits of autografts, this approach is accompanied by several pitfalls such as 43.1% bone resorption in one year [23], limited availability, and significant morbidity [12]; accordingly several measures have been taken to either reduce the amount of harvested bone, decrease secondary resorption [20], or develop a novel technique to eliminate the need for bone grafting. Application of regenerative techniques, either through cells or growth factors, to enhance the healing capacity of the body or decrease donor site morbidities, has been a gateway for bringing tissue engineering principles from the bench to the bedside [22]. Scaffolds have been conventionally administered as a filler or bone substitute in surgeries [24, 25], but in the present study we coapplied scaffolds and MSCs to benefit from osteoinductive, osteoconductive, and osteogenic properties of each of these elements simultaneously. This approach was based on the evidences provided by the previous literature demonstrating increased bone formation in the coapplication of MSCs with scaffolds [26–28] as bone formation was 65.78% once the cells were simultaneously applied with scaffolds comparing to 36.84% in scaffold only group [26] and also in the other study bone formation was 48.63% and 17.27% in MSC+scaffold versus scaffold only, respectively [29]. In the clinical cases using BMMSCs loaded on hydroxyapatite (HA) or hydroxyapatite/beta-tricalcium phosphate (HA/*β*-TCP) also has shown acceptable results in maxillary sinus augmentation [24, 30].

A large body of the literature in tissue engineering has focused on application of BMMSCs in alveolar clefts [31], but some studies have demonstrated that adipose tissue yields 100–500 times greater number of cells than bone marrow aspirates [32–34] with comparable properties comparing to BMMSCs [16, 17]. Several in vitro and in vivo studies support the osteogenic potential of ADSCs [17, 35, 36]; they yield greater number of stem cells in a predefined volume [37], greater proliferation [38], and fibroblastoid colony forming unit (CFU-f) formation [17], and also they have less senescence in vitro [17] and lower malignant transformation [39] in comparison with BMMSCs. Comparison of in vitro osteogenesis of various MSCs on poly (L-lactide) acid (PLLA) scaffold has demonstrated similar ALP and calcium content in BFSCs and BMMSCs [40] and their potential in treatment of periodontal defects is suggested [41, 42]. In the present study we used BFP derived MSCs relying on the literature regarding their osteogenic potential and also due to minimal morbidity and ease of procedure of harvesting BFP [19]. In addition, the stem cell niche theory suggests enhanced bone formation once stem cells are obtained from craniofacial region [43].

Maxillary alveolar cleft defects behave like critically sized defects in the healing sequence. Using autologous bone grafts, alveolar cleft osteoplasty is the standard treatment for the management of alveolar clefts [44]. Pradel et al. demonstrated the successful use of differentiated osteogenic cells for cleft repair in a case report, concluding that the method can lead to spontaneous tooth eruption on the cleft side [3]. In previous experiments, BMMSCs, when cultured over a composite scaffold of demineralized bone and calcium sulfate, showed 24% new bone formation in alveolar cleft defects [13]. Application of MSCs has demonstrated 25% to 79% new bone formation in alveolar cleft defects [15]. The most commonly used growth factor in bone engineering, RhBMP-2, resulted in 65% to 95% bone fill in cleft defects [45, 46]. A combination of BMMSCs and platelet-derived growth factors demonstrated 51% new bone in secondary alveoloplasty in cleft patients [6]. Application of BMMSCs with or without adhesive agents in some critically sized defects demonstrated less than optimal bone formation to heal the defect completely [47]. Using MSCs with the same technique of harvesting and delivery, when cultured over biphasic HA/TCP and mixed with the platelet rich in growth factor as an adhesive agent, resulted in increased bone formation of up to 50% [6]. It is assumed that synthetic or allogenic scaffolds with a structure similar to natural bone may be appropriate replacements for natural human bone and the literature suggests that freeze dried allografts are more osteoinductive than *β*-TCP [48]. In addition NBBM's biocompatibility as a cell carrier was further evaluated in this study.

As discussed earlier, it is assumed that coapplication of MSCs and scaffolds may have synergic effect in bone regeneration and it is well known that autogenous bone is a rich source of cells and growth factors which has made it the gold standard of treatment [49] as lateral ramus cortical bone is one of the most common intraoral donor sites used to treat atrophic ridge in reconstructive surgery [9] and iliac crest is the most commonly used extraoral donor site [8].

However, the secondary resorption associated with grafts casts a doubt in their long term efficacy [50]. In the present study, we tested the hypotheses whether combining autogenous bone grafts with the dual combination of MSC+NBBM may be beneficial in enhancing bone regeneration as well decreasing secondary bone resorption by creating a protective healing barrier over the graft. In the present study, LRCP was used in 3 patients to create a protected healing space fixed between the nasal floor and the buccal and palatal side of the cleft defect. Using LRCP as an autogenous barrier containing MSCs increased the rate of new bone formation and resulted in 69% to 85% BF. One of the patients in the LRCP+BFSC group developed a partial dehiscence and demonstrated only 69% BF. All members of this group were adults (aged 20 to 29 years) and had lower regenerative capacities.

In the AIC+MSC group, the range of BF was between 75% and 90%, higher than that seen using AIC alone (controls), where it was 65% to 85%, although tissue-engineered bone has not shown a high level of evidence for treatment of critically sized defects [15]. In our previous study, in reconstruction of atrophic ridges the autogenous block bones of iliac crest were covered with NBBMs loaded with BFPSCs and the results demonstrated 3.94 mm and 3.01 mm bone gain in the BFPSC+NBBM and control groups, respectively [20]. We assume that complex of MSC and scaffold will further enhance bone regeneration so it may negate and/or compensate the resorption of the graft. Despite the limitations of the present study, such as small sample size and lack of growth factor delivery and lack of a control group with only LRCP grafting, it is demonstrated that applying regenerative techniques with autogenous donor sites seems to enhance bone formation capacity in the alveolar cleft defects and can decrease the amount of harvested autogenous bone. This concept can decrease donor site morbidities and time of hospitalization. Novel structures of scaffolds, such as incorporated canals into the structure with sustained release of growth factors [51], and also new generation of stem cells, such as induced pluripotent stem cells [52], may assist in enhancing the outcomes of bone tissue engineering in clinical setting.

## 5. Conclusion

Combination of MSCs with AIC bone can enhance new bone formation in alveolar cleft bony defects. Intraoral donor sites such as LRCP may be used as a cage to protect scaffolds loaded with MSCs; however future studies with greater samples, and comparison of outcomes in various combination of cells, scaffolds, and growth factors, are encouraged to further pave the way for application of tissue engineering in cleft defects.

## Figures and Tables

**Table 1:** Comparison of mean bone formation between groups.

Group	Number	Defect mm^3^ [mean ± SD]	Bone fill% [mean ± SD]	Min (bone fill%)	Max (bone fill%)
AIC	3	6.80 ± 2.41	70 ± 10.40	65	85
MSCs+LRCP	3	7.99 ± 2.50	75 ± 3.5	69	85
MSCs+AIC	4	7.52 ± 2.91	82.5 ± 6.45	75	90
